# Are Molecular Haplotypes Worth the Time and Expense? A Cost-Effective Method for Applying Molecular Haplotypes

**DOI:** 10.1371/journal.pgen.0020127

**Published:** 2006-08-18

**Authors:** Mark A Levenstien, Jürg Ott, Derek Gordon

**Affiliations:** 1 Laboratory of Statistical Genetics, Rockefeller University, New York, New York, United States of America; 2 Department of Genetics, Rutgers University, Piscataway, New Jersey, United States of America; University of Michigan, United States of America

## Abstract

Because current molecular haplotyping methods are expensive and not amenable to automation, many researchers rely on statistical methods to infer haplotype pairs from multilocus genotypes, and subsequently treat these inferred haplotype pairs as observations. These procedures are prone to haplotype misclassification. We examine the effect of these misclassification errors on the false-positive rate and power for two association tests. These tests include the standard likelihood ratio test (LRT_std_) and a likelihood ratio test that employs a double-sampling approach to allow for the misclassification inherent in the haplotype inference procedure (LRT_ae_). We aim to determine the cost–benefit relationship of increasing the proportion of individuals with molecular haplotype measurements in addition to genotypes to raise the power gain of the LRT_ae_ over the LRT_std_. This analysis should provide a guideline for determining the minimum number of molecular haplotypes required for desired power. Our simulations under the null hypothesis of equal haplotype frequencies in cases and controls indicate that (1) for each statistic, permutation methods maintain the correct type I error; (2) specific multilocus genotypes that are misclassified as the incorrect haplotype pair are consistently misclassified throughout each entire dataset; and (3) our simulations under the alternative hypothesis showed a significant power gain for the LRT_ae_ over the LRT_std_ for a subset of the parameter settings. Permutation methods should be used exclusively to determine significance for each statistic. For fixed cost, the power gain of the LRT_ae_ over the LRT_std_ varied depending on the relative costs of genotyping, molecular haplotyping, and phenotyping. The LRT_ae_ showed the greatest benefit over the LRT_std_ when the cost of phenotyping was very high relative to the cost of genotyping. This situation is likely to occur in a replication study as opposed to a whole-genome association study.

## Synopsis

Localizing genes for complex genetic diseases presents a major challenge. Recent technological advances such as genotyping arrays containing hundreds of thousands of genomic “landmarks,” and databases cataloging these “landmarks” and the levels of correlation between them, have aided in these endeavors. To utilize these resources most effectively, many researchers employ a gene-mapping technique called haplotype-based association in order to examine the variation present at multiple genomic sites jointly for a role in and/or an association with the disease state. Although methods that determine haplotype pairs directly by biological assays are currently available, they rarely are used due to their expense and incongruity to automation. Statistical methods provide an inexpensive, relatively accurate means to determine haplotype pairs. However, these statistical methods can provide erroneous results. In this article, the authors compare a standard statistical method for performing a haplotype-based association test with a method that accounts for the misclassification of haplotype pairs as part of the test. Under a number of feasible scenarios, the performance of the new test exceeded that of the standard test.

## Introduction

With the advent of the HAPMAP project [1,2], the popularity of haplotype-based case-control genetic association studies has grown markedly. The alleles present at multiple genetic markers across a given chromosome form a haplotype [3]. It has been suggested that association studies utilizing haplotypes formed from single nucleotide polymorphisms (SNPs) may be more powerful than single locus association [4–11].

Methods for explicit determination of phased haplotypes are available [12–18]. However, in practice, phased haplotypes are rarely determined explicitly. Instead statistical methods for gene mapping estimate haplotype frequencies from multilocus genotype data [19–28]. For case-control association studies, the sampling design involves unrelated individuals, and therefore the procedure used to estimate haplotype frequencies treats each individual as an independent observation. As with other procedures of statistical estimation, the accuracy of haplotype frequency estimates depends on several factors including “sample size, number of loci studied, allele frequencies, and locus-specific allelic departures from Hardy-Weinberg and linkage equilibrium” [29]. Furthermore, these factors also affect the accuracy of phased-haplotype inference or phased-haplotype calls [30]. Several researchers have investigated the accuracy of haplotype inference procedures by applying them to real and simulated datasets [18,26,30–37].

Several statistical methods are available to perform tests of haplotype-based case-control association. One method calculates the likelihood of the data in terms of the estimated haplotype frequencies. An alternative method relies on the use of a contingency table containing the case-control counts for each inferred haplotype. The counts in the contingency table can be determined either by inferring phased haplotypes for each individual or by multiplying each haplotype frequency estimate by the total number of haplotypes in the study. Many researchers find the latter method appealing since it applies the same format as the classic genotypic and allelic case-control studies, and explicitly accounts for each phased haplotype. As a result, many researchers employ this method in practice [18,35,38–40]. In the event that all phased haplotypes have been called correctly, this method can provide additional power [41,42]. This situation is analogous to tests of association using allele estimates from individual genotypes as compared with allele frequency estimates from DNA-pooling data [43].

However, misclassifications can lower a study's power and/or affect the false-positive rate. The act of calling haplotype pairs from multilocus genotypes in the phase-ambiguous situation is similar to the act of dichotomizing continuous measures. Royston et al. document a loss in power when dichotomizing continuous predictor variables in a regression analysis [44]. In the context of our study, a misclassification results when the haplotype pair called for an individual is not the true underlying haplotype pair. Non-differential misclassification occurs when the misclassification rates are the same in cases and controls. When non-differential misclassification exists, the test suffers a loss in power, but the false-positive rate remains unchanged [45,46]. In contrast, differential misclassification inflates the test's false-positive rate and may diminish its power [47]. We conjecture that in the absence of differential genotype misclassification, all haplotype misclassification is non-differential when haplotype frequency distributions are the same in cases and controls, i.e., under the null hypothesis.

Although there have been several studies aimed at evaluating the accuracy of haplotype inference and haplotype frequency estimation procedures [26,29,30,32,35,37], to our knowledge, no systematic study of the effects of haplotype misclassification has been documented. Thus, the purpose of this work is to address the effects of haplotype misclassification on the false-positive rate and power of commonly used tests of haplotype-based association. Specifically, this research aims to (1) classify the nature of the misclassification present in calling phased haplotypes; (2) determine the appropriateness of using the asymptotic χ^2^ distribution and permutation methods to evaluate the significance of the test statistics we employ; and (3) compare the power of our test statistic which accounts for haplotype misclassification with the power of the standard likelihood ratio test statistic when the costs are fixed.

## Methods

### Test Statistics

In order to detect an association between a haplotype pair and disease status, we employed two statistical tests on 2 × *n* contingency tables where *n* is the number of haplotype pair categories found by inference. These tests include the standard likelihood ratio test (LRT_std_) and a likelihood ratio test that employs a double-sampling approach to allow for the misclassification inherent in the haplotype inference procedure (LRT_ae_). The LRT_std_ is a likelihood ratio statistic that treats the called haplotype pairs as observations, and as a result, the likelihood is the multinomial distribution where the called haplotype pairs are the categories [48]. The LRT_ae_ statistic is a likelihood ratio statistic that employs a double-sampling procedure to account for the misclassification present in haplotype inference. On all the individuals in the study, there is a fallible measure [49,50], the haplotype pairs inferred from the multilocus genotypes, and on a subset of these individuals, there is a second measure that is considered to be infallible [49,50], molecular haplotypes. By comparing the fallible data with infallible data, the LRT_ae_ procedure estimates the misclassification rates present in the fallible data and incorporates this information into the likelihood calculation [51]. The details regarding the LRT_std_ and LRT_ae_ statistics including notation and computation are provided in Protocol S1.

### Permuted and Asymptotic *p*-Values

We applied two methods for evaluating the *p*-value or statistical significance of each statistic. The first method relies on using the central χ^2^ distribution to find the *p*-value since, according to statistical theory under the null hypothesis of no association, twice the natural logarithm of the likelihood ratio follows the central χ^2^ distribution asymptotically for large sample sizes [3,48]. In addition, it has been shown that when Cochran's rule is followed (more than five observations in each cell of the contingency table), the presence of non-differential misclassification does not affect the distribution of the likelihood ratio test statistics under the null hypothesis of no association [46,51]. The second method employs permutation testing to generate the distribution of the test statistic under the null hypothesis and to determine its statistical significance. In this article, *p*-values found with the former and latter approaches are referred to as asymptotic *p*-values and permutation *p*-values, respectively.

### Description of Data Generation and Analysis

**Figure 1:**
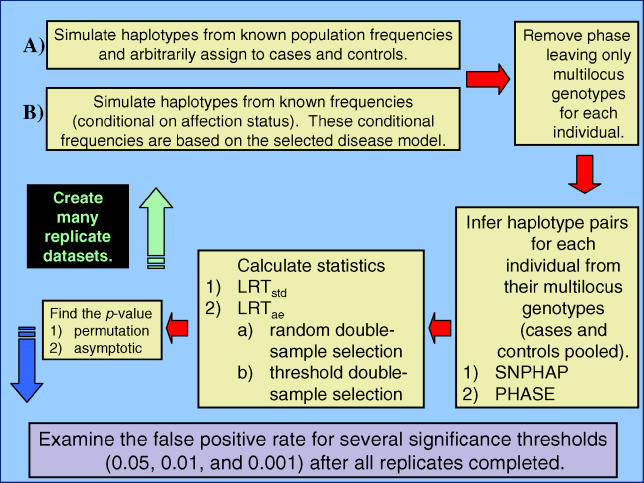
Schematic Flow Chart Illustrating the Procedure Employed for Computing Type I Error and Power by Way of Data Simulation (A) shows type I error, and (B) shows power by way of data simulation.

To investigate the behavior of these test statistics for a variety of situations, we applied these statistical tests to many simulated datasets. Figure 1 illustrates the procedure we used to simulate the data and to evaluate the false-positive rate or type I error and power at fixed significance levels for each statistic. For the analysis of each replicate dataset simulated, the multilocus genotype data from cases and controls were pooled to infer haplotype pairs for each individual. These inferred haplotypes are sufficient for the computation of LRT_std_; however, LRT_ae_ requires additional information in the form of molecular haplotypes for a subset of the individuals in the study. Two alternative procedures for selecting individuals for the double sample (individuals with molecular haplotypes in addition to genotypes) were employed. In one selection scheme, individuals were selected randomly. In the other selection scheme, individuals possessing the most ambiguity in their statistically inferred haplotype pairs were prioritized in selecting the double sample. Specifically, we double-sampled those individuals with the smallest posterior probabilities associated with their inferred haplotype pair up to a posterior probability threshold, *δ,* of 0.85 or until the number of individuals specified by the maximum double-sample proportion was reached. Therefore, under this second scheme, the number of individuals double-sampled varied between replicate datasets. In this article, the former and latter procedures for determined the double sample are referred to as random and threshold double-sample selection, respectively.

### Two-SNP Scenario

#### Evaluation of false-positive rate for permutation and asymptotic *p*-values.

For the simplest non-trivial case, the scenario in which the haplotype under evaluation includes two SNPs, we applied a fractional factorial design [52] to perform a comprehensive study of type I error. For the type I error, haplotype pairs were inferred using both SNPHAP v 1.3.1 (see Electronic Database Information) and PHASE v 2.1.1 [27] (see also Electronic Database Information). Table 1 contains the fractional factorial design settings for the study of type I error for the scenario involving two SNP markers. We consider a 1/2(2*^k^*) fractional factorial design, where *k* = 6. Because of redundancy, we were able to reduce the number of experimental runs from 32 to 18. For instance, under the null hypothesis of no association, a run with 1,000 cases and 250 controls is equivalent to a run with 250 cases and 1,000 controls (with all other factors having equal settings to those for the first run). During each run, 10,000 replicate datasets were simulated. We performed the 18 runs with both of the two alternative procedures for selecting the double sample: random and threshold double-sample selection. For the threshold double-sample selection method, *δ* was 0.85, and the maximum double-sample proportion was set to the value of *α* in the fractional factorial design.

**Table 1:**
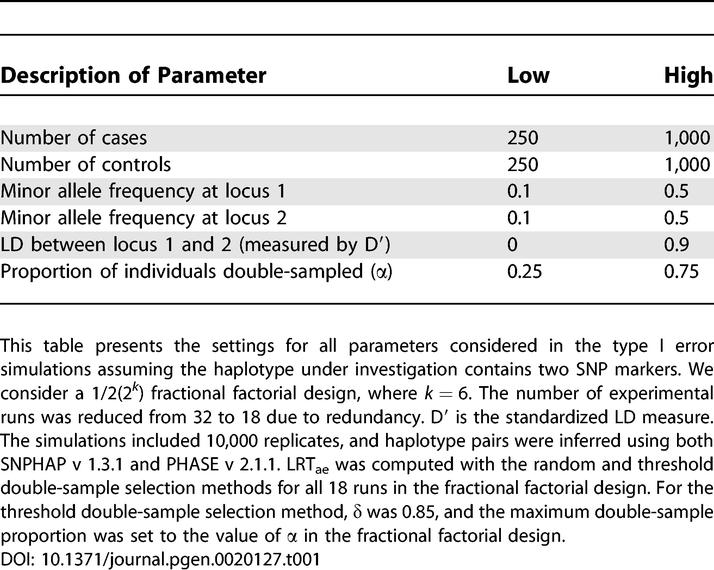
Fractional Factorial Design Parameter Settings for the Study of Type I Error Assuming the Haplotype under Investigation Contains Two SNP Markers

To evaluate each test statistic's ability to maintain the correct type I error, we examined the distribution of the *p*-values computed for data simulated under the null hypothesis of no association. We performed two goodness-of-fit tests, the Kolmogorov-Smirnov (KS) and the Anderson-Darling (AD) tests [53] to determine whether the *p*-values deviate significantly from the standard uniform distribution, and examined the false-positive rate for significant thresholds of 0.05, 0.01, and 0.001.

#### Evaluation of power for fixed cost.

We also evaluated the behavior of these statistics under the hypothesis that an unobserved disease locus exists in linkage disequilibrium (LD) with the haplotype under study. Table 2 contains the factorial design settings for the power study in the scenario involving two SNP markers. The factorial design includes three factors: disease model, genotype relative risk [54] for the homozygote genotype *(R_2_)*, and the disease allele frequency *(DAF)*. Each factor contains two levels. For the disease model factor, the two levels are a dominant disease model and a multiplicative disease model. The dominant disease model requires that *R_2_ = R_1_* whereas the multiplicative disease model requires that *R_2_ = R_1_^2^*, where *R_1_* and *R_2_* are the genotype relative risks for the heterozygote and homozygote genotypes, respectively. Specifically, the genotype relative risks are defined as the following. If the penetrances, *f_i_,* are defined by *f_i_*=Pr(affected|*i* copies of disease allele), where *i* = 0, 1, or 2, the genotype relative risks, *R_1_* and *R_2_*, are defined by *R*_1_=*f*_1_/*f*_0_and *R*_2_=*f*_2_/*f*_0_, respectively [54].

**Table 2:**
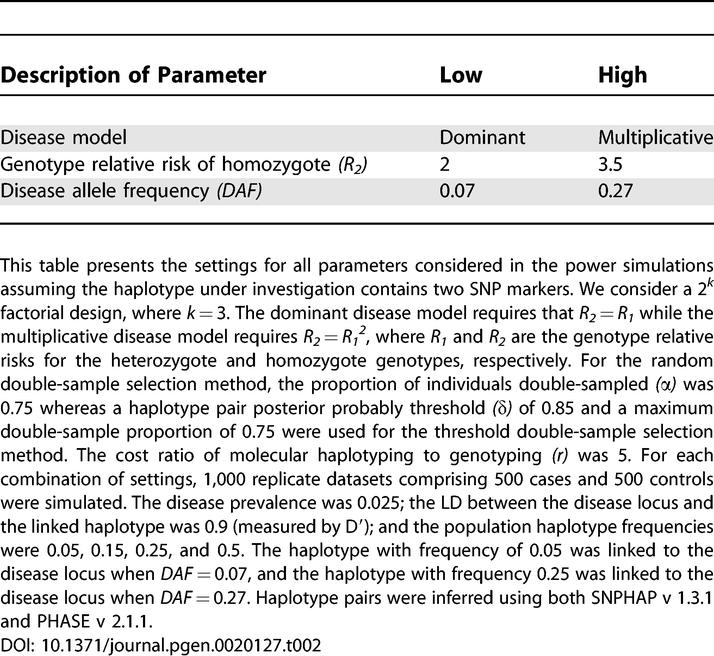
Factorial Design Parameter Settings for the Study of Power Assuming the Haplotype under Investigation Contains Two SNP Markers

As with the study of type I error, we inferred the haplotypes for the power simulations with both SNPHAP v 1.3.1 and PHASE v 2.1.1. The proportion of individuals double-sampled, α, for the LRT_ae_ method (random double-sample selection) was set at 0.75. For the threshold double-sample selection, *δ* was set to 0.85, and the maximum double-sample proportion was 0.75. In the power simulations, the conditional haplotype frequencies were found from the specified disease model parameters by the method described previously [7,55] (also see the PAWE Web site at http://linkage.rockefeller.edu/derek/pawe1.html). However, we selected a specific haplotype to be in LD with the disease locus. During each run, 1,000 replicate datasets consisting of 500 cases and 500 controls were simulated. For these simulations, the disease prevalence was 0.025; the LD between the disease locus and the linked haplotype was 0.9 (measured by D′ [56]); and the population haplotype frequencies were 0.05, 0.15, 0.25, and 0.55. The selection of the specific haplotype in LD with the disease locus depended on the *DAF*. The haplotype occurring with a frequency most similar to that of the disease allele was selected. Thus, the haplotypes with frequencies of 0.05 and 0.25 were selected as the variant in LD with the disease when the *DAF* was set at 0.07 and 0.27, respectively. As with the evaluation of the false-positive rate, we performed all eight runs from the factorial design using both random and threshold double-sample selection.

To compare the power of the two test statistics, we evaluated the power of the statistics under fixed cost conditions. Since the LRT_ae_ requires the additional cost associated with obtaining molecular haplotypes on a subset of the samples, we reduced the number of samples when the LRT_ae_ statistic was applied so that the same total cost would be incurred as for the runs with the LRT_std_. The reduced sample size for the LRT_ae_ sample was computed using Equation 1,

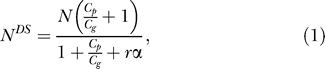
where *N^DS^* is the sample size for the LRT_ae_; *N* is the sample size for the LRT_std_; *C_p_* is the cost of phenotyping, *C_g_* is the cost of genotyping; *r* is the cost ratio of molecular haplotyping to genotyping *(C_mh_/C_g_)*; and *α* is the proportion of individuals in the LRT_ae_ sample that have molecular haplotypes determined (double-sampling proportion). We consider the phenotyping costs, *C_p_*, to include costs associated with ascertainment and diagnosis. We illustrate fixed-costs sample sizes for the following example. With settings of *C_p_/C_g_* = 25, *r* = 5, *α* = 0.75, and *N* = 1,000 for the LRT_std_ method, the corresponding total sample size for the LRT_ae_ method, *N^DS^,* is 874. The reader should note that the reduced sample size results from the additional cost incurred by double-sampling 75% of the total sample for the LRT_ae_ method. If *C_p_/C_g_* = 1,000, note that this term will dominate the expression in  Equation 1, and the fixed-cost sample size, *N^DS^,* will not differ greatly from the sample size for the LRT_std_, *N*. All power simulations were performed under fixed-cost conditions. Since the double-sample proportion, *α,* varies from replicate to replicate when the threshold double-sample selection method is employed, we first performed several test runs to determine the mean double-sample proportion, 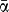 . Using 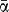 , we computed N^DS^*, the total sample size for the LRT_ae_ determined from the expectation of *α*. Here, the asterisk is added to indicate that total sample size is computed using the expected value of *α,* as compared with the random double-sample selection, in which *α* is a fixed quantity. For a specific disease model, we performed a comprehensive study of the power difference between the LRT_ae_ and LRT_std_ for the situation of a haplotype comprising two SNPs.

### Multi-SNP Scenario

#### Evaluation of false-positive rate and power for fixed costs.

**Figure 2:**
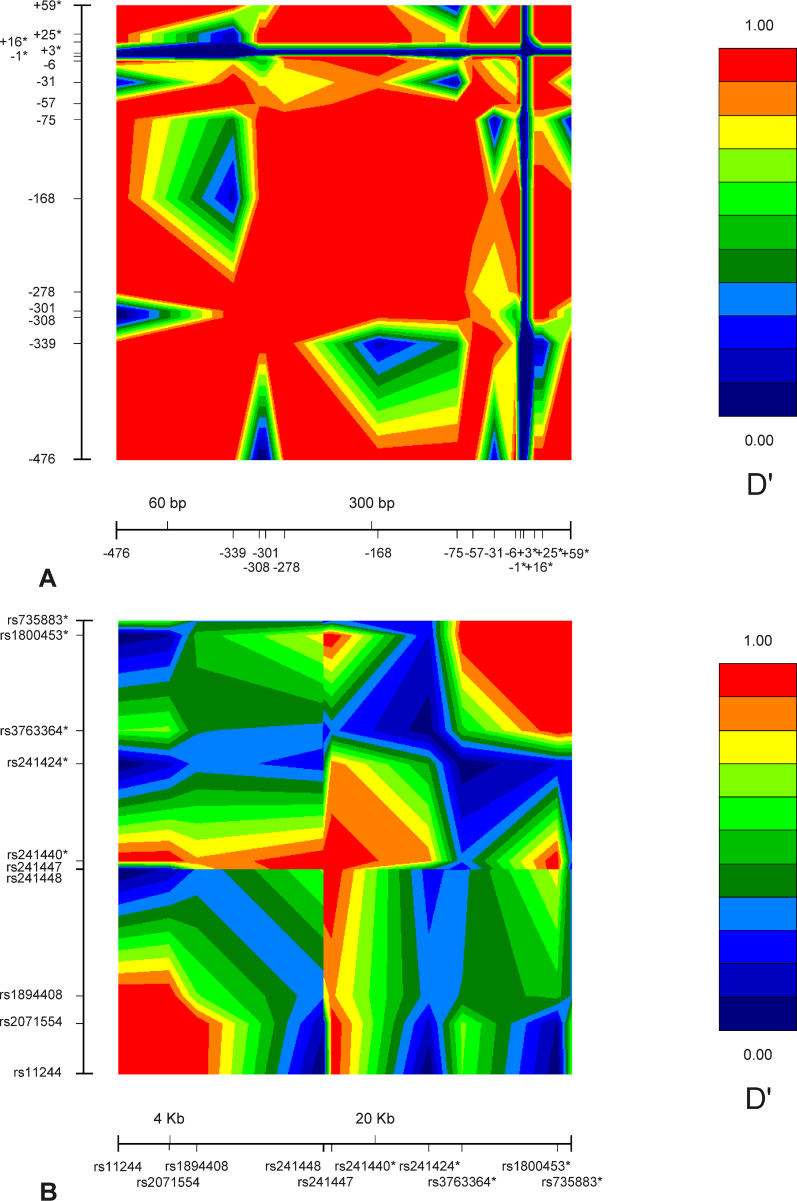
GOLD Plots Showing the Pair-Wise Intermarker LD in Terms of D′ (A) shows the LD for 15 SNP markers within the proximal promoter region of human pituitary expressed growth hormone (GH1), and (B) shows the LD for ten SNP markers within the *TAP2* gene. In (A), the SNP markers are listed as their position relative to the transcription start site of the *GH1* gene whereas in (B), the SNP markers are listed by their National Center for Biotechnology Information (NCBI) reference SNP (rs) numbers. Physical distances are provided. All SNP markers displayed were included in the type I error study whereas only the SNP markers accompanied by an asterisk (*) were included in the power study.

Through additional simulations, we investigated the behavior of these statistics when applied to haplotypes comprising larger numbers of SNPs. Because these simulations required additional computational time, we only used SNPHAP v 1.3.1 (see Electronic Database Information) for inferring haplotypes. Our simulations were based on haplotype frequencies from two datasets: (1) a dataset of molecular haplotypes with very high levels of pair-wise LD between markers [14] and (2) a dataset of multilocus genotypes from the *TAP2* gene within the major histocompatibility complex, a region with low pair-wise LD between markers [1,2] (see also Electronic Database Information), hereafter referred to as the Horan and the HapMap *TAP2* datasets, respectively. Figure 2 displays the inter-marker LD for each of these two datasets using GOLD plots [57]. For the Horan dataset, we determined the generating population haplotype frequencies for our simulations directly using the counting method [3]. For the HapMap *TAP2* dataset, we found the generating population haplotype frequencies for our simulations indirectly using SNPHAP v 1.3.1 (see Electronic Database Information). In the latter case, haplotype frequencies were estimated from the parents of each trio in the Yoruba population group from the International HapMap Project. For the type I error simulation studies, 1,000 replicate datasets containing 250 cases and 250 controls were simulated. For the type I error runs based on the Horan data and the HapMap *TAP2* data, we simulated haplotypes comprising 15 SNPs and ten SNPs, respectively, whereas for the power runs, we simulated haplotypes comprising five SNPs [1,2,14]. Figure 2 specifies the SNPs we used from each of the datasets in the type I error and power runs. For the Horan dataset, we provide the SNP markers' positions (relative to the transcription start site of the *GH1* gene) whereas for the HapMap *TAP2* dataset, we provide the name of the SNP marker. As a result, we simulated haplotypes using 17 haplotype variants with frequencies greater than 0.01 for both the Horan and HapMap *TAP2* type I error simulations. In addition, we simulated haplotypes using five and ten haplotype variants with frequencies greater than 1/(2*s*), where *s* is the total number of individuals, for the Horan and HapMap *TAP2* power simulations, respectively. For each scenario, we normalized the frequencies so that they summed to unity. As with the power studies for the two-SNP scenario, the selection of the specific haplotype in LD with the disease locus depended on the *DAF*. The rationale for the selection procedure is provided in the Results section addressing multi-SNP power. For multi-marker type I error and power studies, we employed both the random and threshold double-sample selection methods in computing the LRT_ae_ statistic. When the random double-sample selection method was used, the double-sample proportion, *α,* was 0.75. When the threshold double-sample method was used, the setting of *δ* = 0.85 was used, and the maximum proportion of individuals included in the double-sample was 0.75.

#### Identifying the nature of haplotype pair misclassification.

For all the simulations performed, we recorded the details of the misclassifications that occurred. Specifically, for every replicate, we computed the misclassification rates,

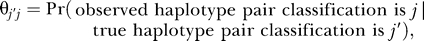
where *j*′≠*j* are integers ranging from 1 to the maximum number of haplotype pairs [51]. Previous research studying genotype misclassification rates in tests of genotypic association provides the motivation for ascertaining these values [58,59]. This notation is also used in Protocol S1.

## Results

### Two-SNP Scenario

Our results for type I error and power were almost identical from the simulations utilizing SNPHAP v 1.3.1 and PHASE v 2.1.1 for the haplotype inference. Although we present graphs and tables that display the results provided by SNPHAP v 1.3.1 for the haplotype inference, the reader should note that similar results were found using PHASE v 2.1.1.

#### Evaluation of false-positive rates for permutation and asymptotic *p*-values.

**Figure 3:**
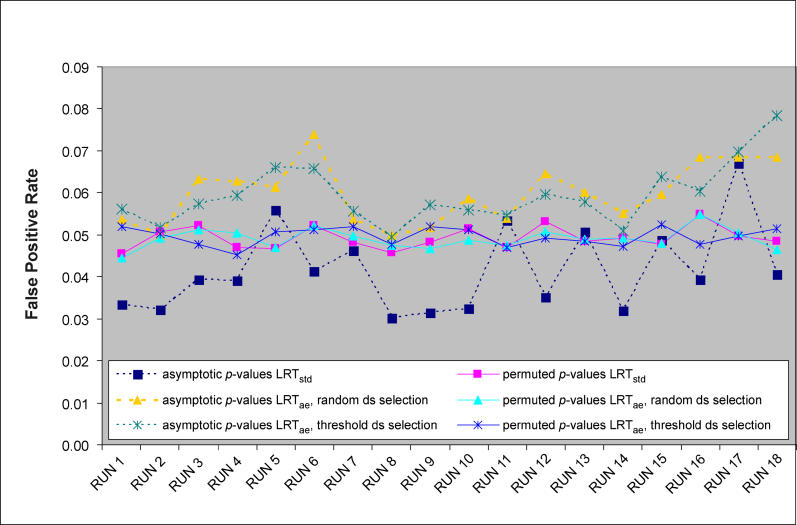
Line Graph Illustrating Estimates of the False-Positive Rate at the 0.05 Significance Level for LRT_std_ and LRT_ae_ The *p*-values were determined by both permutation and the asymptotic central χ^2^ distribution. The 18 runs correspond to the combinations of parameter settings described in Table 1. For all 18 runs, LRT_ae_ was computed with the random and threshold double-sample selection methods. When the threshold double-sample method was used to compute LRT_ae_, the setting of *δ* = 0.85 was used, and the maximum proportion of individuals included in the double sample was the value for *α* specified by the fractional factorial design. SNPHAP v 1.3.1 was used for the haplotype inference for the simulation results displayed in the graph.

The type I error simulations demonstrated that the approach for determining statistical significance is critical for maintaining the correct false-positive rate. Although the KS and Anderson-Darling (AD) test results indicated that the distribution of permutation *p*-values was consistent with the standard uniform distribution, they also indicated that the distributions of asymptotic *p*-values did not resemble the standard uniform distribution. These results were reinforced by the false-positive rates we found. For all the simulation runs displayed in Table 1, Figure 3 shows the false-positive rate for a significance threshold of 0.05 for LRT_std_ and LRT_ae_ (using the random and threshold double-sample selection methods) association tests in which statistical significance was indicated by permutation and asymptotic *p*-values. The graph shows that asymptotic *p*-values for LRT_ae_ are anti-conservative whereas those for LRT_std_ fluctuate between conservative and anti-conservative values. In contrast, the permutation *p*-values for both statistics consistently maintain the nominal significance level of 0.05. We found that the asymptotic and permuted *p*-values demonstrated similar behavior for significance thresholds of 0.01 and 0.001 (unpublished data). SNPHAP v 1.3.1 was used for the haplotype inference for the simulation results displayed in the graph. These results are not surprising since several simulation parameter settings have expected cell counts of less than five counts, violating Cochran's rule [60].

#### Evaluation of power for fixed cost.

Based on the results for the false-positive rates, we conclude that power can only be evaluated using the permutation *p*-values. We compare the power of LRT_ae_ (using the random and threshold double-sample selection methods) to LRT_std_. Table 3 presents summary statistics for the power difference (LRT_ae_ power − LRT_std_ power) at various significance levels for the two cost ratios *C_p_/C_g_* = 25 and *C_p_/C_g_* = 1,000 using the eight parameter settings from the factorial design (Table 2). Note that in all runs, we set the cost ratio of molecular haplotyping to genotyping, *r,* to be 5, and the proportion of individuals to be double-sampled, α, to be 0.75 (for the random double-sample selection method). The values reported correspond to the simulations utilizing SNPHAP v 1.3.1.

**Table 3:**
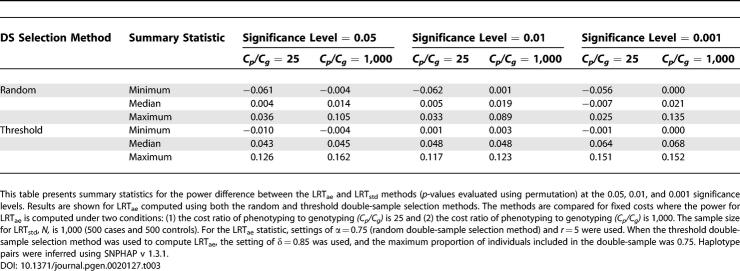
Summary Statistics for Power Difference (LRT_ae_ − LRT_std_) at Various Significance Levels

For the random double-sample selection method, the minimum power difference observed occurred when *C_p_/C_g_* = 25 for a dominant disease model with *R_2_* = 2 and *DAF* = 0.27 at a significance level of 0.01. For these settings, the LRT_ae_ power was 0.544 and LRT_std_ power was 0.606. The maximum power difference observed occurred when *C_p_/C_g_* = 1,000 for a dominant disease model with *R_2_* = 3.5 and *DAF* = 0.07 at a significance level of 0.001. For these settings, the LRT_ae_ power was 0.910 and LRT_std_ power was 0.775.

For the threshold double-sample selection method, the minimum power difference observed occurred when *C_p_/C_g_* = 25 for a dominant disease model with *R_2_* = 2 and *DAF* = 0.27 at a significance level of 0.05. For these settings, the LRT_ae_ power was 0.821 and LRT_std_ power was 0.831. The maximum power difference observed occurred when *C_p_/C_g_* = 1,000 for a dominant disease model with *R_2_* = 2 and *DAF* = 0.07 at a significance level of 0.05. For these settings, the LRT_ae_ power was 0.573 and LRT_std_ power was 0.411.

#### Power difference as a function of double-sample proportion and cost ratio.

**Figure 4:**
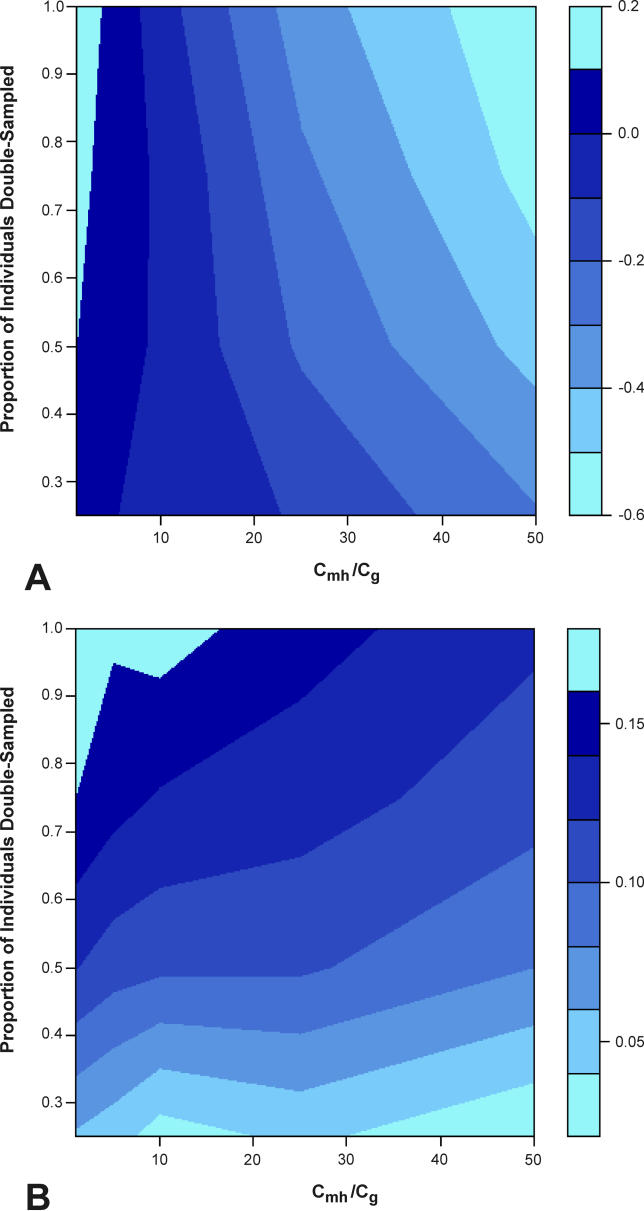
Contour Plots of the Power Difference between the LRT_ae_ and LRT_std_ Methods at Various Settings Various settings for the cost ratio of molecular haplotyping to genotyping *(r)* and the proportion of individuals double-sampled *(α)* are shown. Power is compared at the 0.001 significance level. The cost ratio of phenotyping to genotyping for (A) is 25 while the cost ratio of phenotyping to genotyping for (B) is 1,000. The generation of haplotype frequencies for the cases and controls was based on a dominant disease model with *R_2_* = 3.5 and DAF = 0.07, as well as population haplotype frequencies of 0.05, 0.25, 0.15, and 0.55. The haplotype with frequency of 0.05 was placed in LD (D′ = 0.9) with the disease locus. LRT_ae_ was computed with the random double-sample selection method only. Haplotype pairs were inferred using SNPHAP v 1.3.1.

In the spirit of response surface analysis for factorial design [52], we performed a more thorough analysis of the parameter settings that provided the maximum power difference with LRT_ae_ computed with the random double-sample selection method. These parameter settings are a dominant disease model with *R_2_* = 3.5 and *DAF* = 0.07. These setting provided the additional benefit of power results greater than 75% for both the LRT_ae_ and LRT_std_ methods at the 0.05, 0.01, and 0.001 significance levels for both cost ratios of *C_p_/C_g_* = 25 and *C_p_/C_g_* = 1,000. The analysis involved computation of the LRT_ae_ with the random double-sample selection method. Figure 4 displays the two-dimensional contour plot of the power difference between the LRT_ae_ and the LRT_std_ as a function of *r,* the cost ratio of molecular haplotyping to genotyping, and *α,* the proportion of individuals double-sampled. These power differences are computed for the fixed parameter settings of *C_p_/C_g_* = 25 (Figure 4A) and *C_p_/C_g_* = 1,000 (Figure 4B) at significance level = 0.001 for the disease model described immediately above. The values of *r* considered in the contour plots are 1, 5, 10, 25, and 50 whereas the values of *α* considered are 0.25, 0.50, 0.75, and 1.0. One should note that *α* = 1.0 indicates that all individuals in the study are double-sampled regardless of phase ambiguity. Simulations were performed with 1,000 replicates and 10,000 permutations for each combination of parameters, and SNPHAP v 1.3.1 was used for the haplotype inference. The sample size for the LRT_std_, *N,* was 1,000 (equal numbers of cases and controls). Figure 4A shows that the LRT_ae_ provides a power advantage over the LRT_std_ when *r* is less than 10 and *α* is greater than 0.5. The maximum power gain is 0.16 and occurs when *r* and *α* are 1.0. Conversely, when the *r* is greater than 10, LRT_ae_ is less powerful than LRT_std_ for these parameter settings. The maximum power loss is 0.58 and occurs when *r* is 50 and *α* is 1.0. Note that for these values, the total sample available for the LRT_ae_ method, *N^DS^* (Equation 1), is 342 whereas the total sample available for the LRT_std_ method, *N,* is 1,000.

Figure 4B illustrates that LRT_ae_ is always at least as powerful as the LRT_std_ when *C_p_/C_g_* = 1,000. We observe the minimum power gain of 0.02 when *r* is 50 and *α* is 0.25 and the maximum power gain of 0.17 when *r* and *α* are 1.0. Furthermore, Figure 4B indicates that for any cost ratio, *r,* increasing the double-sampling proportion, *α,* always increases the power gain with the maximum power gain occurring when *α* = 1.0.

### Multi-SNP Scenario

#### Evaluation of false-positive rates for permutation and asymptotic *p*-values.

Table 4 displays our estimates of the false-positive rates using a significance threshold of 0.05 and the results of the KS test for the Horan and HapMap *TAP2* dataset-based simulations. Again, only the permuted *p*-values resemble the standard uniform distribution. In addition, the permuted *p*-values maintained the nominal significance level whereas the asymptotic *p*-values are anti-conservative. The false-positive rate estimates for significance thresholds of 0.01 and 0.001 displayed similar characteristics (unpublished data).

**Table 4:**
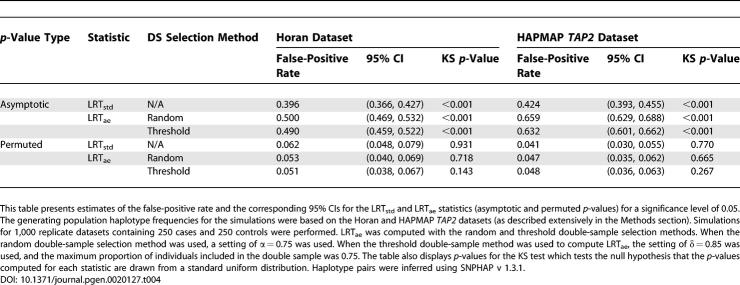
False-Positive Rate Estimates for Simulations with Generating Population Haplotype Frequencies Based on the Horan and HAPMAP *TAP2* Datasets

#### Evaluation of power for fixed cost.

In our power study for haplotypes comprising five SNPs, we again used the disease model parameter settings that provided the maximum power difference (LRT_ae_ power − LRT_std_ power) for the two-SNP factorial design (Table 2) with LRT_ae_ computed using random double-sample selection. These parameter settings are a dominant disease model with *R_2_* = 3.5 and *DAF* = 0.07. We based the population haplotype frequencies on the Horan and HapMap *TAP2* datasets as described in the Methods section. For each dataset, we selected the haplotype with frequency closest to 0.05 as the haplotype in LD with the disease locus. By this choice of haplotype, we approximated the frequency of the linked haplotype for the two-SNP scenario (see Material and Methods section) when *DAF* = 0.07. As with the two-SNP power study, the LD between the disease locus and the linked haplotype was 0.9 (measured by D′) [56]. The cost ratio of molecular haplotyping to genotyping *(r)* was 5. When the random double-sample selection method was used to compute LRT_ae_, the double-sample proportion *(α)* was 0.75. When the threshold double-sample method was used to compute LRT_ae_, the setting of *δ* = 0.85 was used, and the maximum proportion of individuals included in the double-sample was 0.75.

For the Horan dataset, the power estimates for the LRT_std_ and the LRT_ae_ were almost identical at the 0.05, 0.01, and 0.001 significance levels for cost ratios *(C_p_/C_g_)* of both 1,000 and 25 (unpublished data). The high pair-wise intermarker LD present in the Horan dataset causes the haplotype inference to occur with almost complete fidelity. In the absence of misclassification, the LRT_ae_ statistic reduces to the LRT_std_. Therefore, the high degree of similarity in power for these statistics is not surprising.

For the HAPMAP *TAP2* dataset, Table 5 displays the power estimates and the corresponding 95% confidence intervals (CIs) for the LRT_std_ and LRT_ae_ methods at the 0.05, 0.01, and 0.001 significance levels assuming fixed costs. When *C_p_/C_g_* = 1,000, the LRT_ae_ provides a substantial power benefit over the LRT_std_ with the power difference ranging from 6% and 7% at a significance level of 0.05, to 14% and 21% at a significance level of 0.001 for random double-sample selection and threshold double-sample selection, respectively. When *C_p_/C_g_* = 25, the advantage of the LRT_ae_ over the LRT_std_ is still substantial for threshold double-sample selection, but more modest for random double-sample selection. For the three significance levels under investigation, the power difference ranged from 7% to 22%, and 1% to 3.5% for threshold and random double-sample selection, respectively.

**Table 5:**
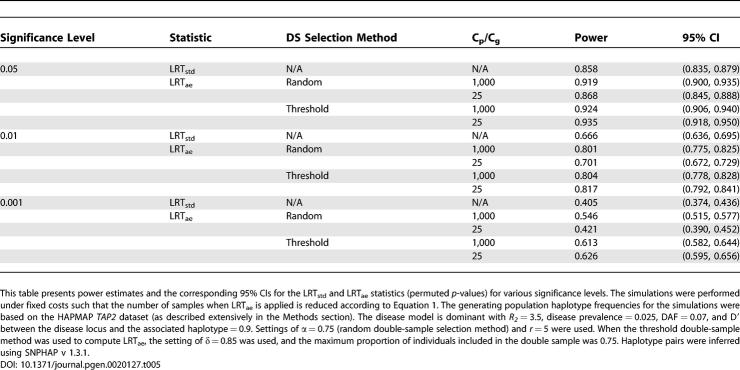
Power Estimates for Simulations with Generating Population Haplotype Frequencies Based on the HAPMAP *TAP2* Datasets

We found that the median power gain of the LRT_ae_ over the LRT_std_ for the threshold double-sample selection method was consistently greater than that for the random double-sample selection method for the runs associated with the factorial design settings displayed in Table 2 and the HAPMAP *TAP2* power simulations (see Tables 3 and 5). Furthermore the power gain for the threshold double-sample selection method occurred for either setting of *C_p_/C_g_*. For the threshold double-sample selection method, 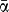 was small (less than 21%) in our simulations so that our computed N^DS^* values had a minimum of 963 individuals.

## Discussion

In practice, few researchers employ molecular haplotyping techniques in genetic case-control studies. The absence of a high-throughput procedure relative to current SNP genotyping technologies is arguably the main reason that this methodology is not more widely used. Another related reason is the cost in terms of both the time and money associated with employing this methodology. Our research suggests that the additional costs involved in molecular haplotyping may be worth the effort, especially if the cost of phenotyping is high relative to the cost of genotyping for a study. Ji et al. found analogous results for the effects of genotype misclassification on genotypic test of association [61]. In practice, this situation arises for replication studies. A genome-wide scan involving thousands of SNP markers along with subsequent fine mapping in an initial set of case and control individuals may identify a number of promising regions for follow-up studies. These follow-up or replication studies involve recruiting an independent sample of cases and controls for which only SNPs in the promising regions will be genotyped [62]. In replication studies for complex traits, the cost ratio of phenotyping to genotyping may be on the order of thousands. For these situations, the LRT_ae_ for testing haplotype association should provide the most utility. It is interesting to note, however, that applying the threshold double-sample selection method provided comparable powers for both high and low phenotyping to genotyping cost ratios. This finding suggests that this selection strategy may provide additional power for an initial genome-wide association study, as well as for a replication study.

One potential limitation of these test statistics that we selected is the increase in degrees of freedom associated with using haplotype pairs rather than individual haplotypes. In general, larger degrees of freedom may result in a loss of power. That is, methods that fully account for uncertainty in the phase-assignment process [11,63,64] may be more powerful than LRT_ae_ because the LRT_ae_ method examines haplotype pairs rather than single haplotypes and therefore has more degrees of freedom. We chose these statistics for the following reasons: (1) The most general misclassification model involves modeling errors in haplotype pairs rather than in individual haplotypes [51,65,66]. (2) When haplotype pair frequencies deviate from Hardy-Weinberg Equilibrium in either case or control sample populations, test statistics that use single haplotype frequencies may increase false-positive rates and/or lose power [67,68]. (3) In contrast with methods that use single haplotype frequencies, the Cochran-Armitage Linear Test of Trend maintains the nominal false-positive rate and does not lose power [68–70]. To our knowledge, a version of this test that incorporates double-sampling procedures to correct for haplotype miscalls does not currently exist.

A point for further research involves identifying the scenarios that produce differential and non-differential haplotype pair misclassification, respectively, as well as identifying the effects of each kind of misclassification on type I error and power. Under the null hypothesis that haplotype frequency distributions are equal in case and control populations, theoretical and simulation studies (including ours in this work) suggest that misclassification is non-differential. Under the alternative hypothesis, it is conceivable that haplotype pair misclassification rates may be different in case and control populations. Although recent research [47,71] indicates that differential misclassification increases the type I error, the effects of differential misclassification on the power of these statistics are unclear.

Although the current perception may be that molecular haplotyping costs are not cost-effective, recent publications suggest that for relatively small regions of the genome, accurate molecular haplotyping is no more expensive than performing fluorescent polymerase chain reactions [18]. In addition, current techniques are able to provide molecular haplotypes for an entire chromosome at a cost ratio *(C_mh_/C_g_)* of approximately 5 (C. Ding, personal communication). Finally, as technology improves, the costs associated with molecular haplotyping will likely decrease, and the throughput will likely increase.

### Conclusion

In this work, our simulations showed that the misclassification present in calling phased haplotypes from multilocus genotypes using statistical methods is complete. That is, each misclassified haplotype pair is consistently misclassified as the same incorrect haplotype pair throughout the entire dataset. In addition, our simulations under the null hypothesis of no association demonstrate that applying the theoretical χ^2^ distribution to evaluate the significance of test statistics produces conservative and anticonservative *p*-values whereas applying permutation methods consistently produces *p*-values that maintain the nominal false-positive rate. Consequently, permutation methods should be exclusively used to determine statistical significance for the tests we perform. As expected, the LRT_ae_ provides the greatest advantage in terms of power over the LRT_std_ in situations in which more haplotype misclassification errors are present. These situations arise when the haplotype under investigation comprises many SNP markers with low pair-wise intermarker LD.

For fixed costs, the power gain of the LRT_ae_ over the LRT_std_ varied depending on the relative costs of genotyping, molecular haplotyping, and phenotyping. In general, the LRT_ae_ showed the greatest benefit over the LRT_std_ when the cost of phenotyping was very high relative to the cost of genotyping. This situation is likely to occur in a candidate gene replication study as opposed to a genome-wide association study. For intermediate phenotyping to genotyping cost ratios (e.g., *C_p_/C_g_* = 25), the LRT_ae_ may still provide a power advantage if the cost ratio of molecular haplotyping to genotyping is low (*C_mh_/C_g_* < 10 for *α* ≥ 0.5). Currently, inexpensive long-range PCR methods for molecular haplotyping are under development. As technology improves leading to less-expensive molecular haplotyping methods, the LRT_ae_ will become applicable to a wider set of circumstances.

### Electronic Database Information

The documentation for SNPHAP and PHASE can be found at http://www-gene.cimr.cam.ac.uk/clayton/software and http://www.stat.washington.edu/stephens/software.html respectively.

The documentation for PAWE can be found at http://linkage.rockefeller.edu/derek/pawe1.html.

Data for the estimation of haplotype frequencies from SNP markers within the *TAP2* gene were downloaded from http://www.hapmap.org/downloads/index.html.en (HapMap public release #16c.1).

LRT_ae_ software is available for free download from ftp://linkage.rockefeller.edu/software/lrtae.

## Supporting Information

Protocol S1 Mathematical Formulation of LRT_ae_ and LRT_std_ Statistics (415 KB DOC)

## Abbreviations

CI: confidence interval
*DAF*: disease allele frequency
KS: Kolmogorov-Smirnov
LD: linkage disequilibrium
LRT_ae_: likelihood ratio test allowing for error
LRT_std_: standard likelihood ratio test
SNP: single nucleotide polymorphism
