# Supplementary material for: Are Molecular Haplotypes Worth the Time and Expense? A Cost-Effective Method for Applying Molecular Haplotypes
Source: PLoS Genet. 2006 Aug 18;2(8):e127. doi: 10.1371/journal.pgen.0020127 (PMC1550282; doi:10.1371/journal.pgen.0020127)
Supplement: Supplementary file 1 [file pgen.0020127.sd001.doc]

# Protocol S1

In this section, we present the mathematical basis for the computation of the LRT_ae_ and LRT_std_ statistics. This work largely follows from our original publication on the LRT_ae_ statistic [51]. The primary difference is that, in this work, we assume only misclassification in haplotype pairs (called “genotypes” in our original publication) and therefore we assume no misclassification of phenotype. Because we do not double-sample on phenotype, we assume that all phenotype classifications are correct. We begin with some notation.

# *Notation*

For all terms, the index is either 0 (case) or 1 (control) and the integer indices range from 1 through *k* inclusive, where *k* is the number of haplotype pairs.

We use prime superscripts to distinguish true categories from observed categories. For example, refers to the true haplotype pair classification for an individual. Also, we use the superscript *t* to denote “true” (as compared with observed) when referring to either an event or a parameter. For example, the notation represents the event that an individual’s true haplotype pair classification is , whereas the notation represents the event that an individual’s observed haplotype pair classification is (see below). Similarly, the notation represents the true probability of the haplotype pair for individuals with (true) phenotype classification , whereas the notation represents the observed probability of the haplotype pair for individuals with phenotype classification . With this notation, we may distinguish between the events and and the probabilities and .

## List of notation

= Number of individuals with (true) phenotype category , true haplotype pair categoryand observed haplotype pair category . (These individuals are double-sampled on haplotype pair classification)

----

.

----

= Number of individuals with (true) phenotype category and observed haplotype pair category .

----

; Note that *n* is the total sample size.

----

=Event that an individual has phenotype .

=Event that an individual has observed haplotype pair .

=Event that an individual has true haplotype pair .

=Event that an individual has phenotype and true haplotype pair .

= True sampling frequency of phenotype .

= Observed population frequency of haplotype pair for individuals with true phenotype .

= True population frequency of haplotype pair for individuals with phenotype .

----

= True population frequency of haplotype pair under the null hypothesis that .

----

= Observed population frequency of haplotype pair under the null hypothesis that .

Note: For each . Also,.

----


# Note: When , these parameters are referred to as misclassification parameters [45,50]. We make use of the double-sample data structure to determine estimates of haplotype pair misclassification values . The misclassification parameter estimates are (see below).

----

= The number of individuals that have been classified by the fallible method as haplotype pair *j* and by the infallible method as haplotype pair .

----

.

----

=Log-likelihood of data as represented in equation (A4), where haplotype pair frequencies are allowed to differ among different phenotype classes. (i.e., is not necessarily equal to for every )

----

=Log-likelihood of data as represented in equation (A4) below, where haplotype pair frequencies are constrained to be equal among different phenotype classes. (i.e., for every )

----

=Log-likelihood of data when not correcting for misclassification, where haplotype pair frequencies are allowed to differ among different phenotype classes. (i.e., is not necessarily equal to for every ) (also see equation A1b below)

----

=Log-likelihood of data when not correcting for misclassification, where haplotype pair frequencies are constrained to be equal among different phenotype classes. (i.e., for every ) (also see equation A1b)

----

## Log-likelihood of observed data and likelihood ratio test statistics

We compute the log-likelihood of the observed data under the null and alternative hypotheses, allowing for error. The null hypothesis we test is for all haplotype pairs . The alternative hypothesis is for at least one . Under either hypothesis, we have, by definition, the log-likelihood of the data given by:

(A1a)

where the notation is the probability of observing event *A* and event *B* and event *C* and so forth and represent the counts for different categories of double-sample information (see above for definitions of all notation). For example, is the number of individuals who have been double-sampled on haplotype pair classification and who have true phenotype classification , true haplotype pair classification , and observed haplotype pair classification . In equation (A1a), the subscript runs over all phenotype classifications () and the subscripts run over all haplotype pair classifications ().

When there are no double-sample data or when we assume that there is no error in the data, equation (A1a) reduces to:

(A1b)

A key assumption in our work is that, conditional on the underlying true data, the observed data are independent. That is, the measurement process for phenotype is independent of the measurement process for haplotype pair classification, so that . It follows that:

(A2)

Using equation (A2) and the fact that

(A3)

we may rewrite the log-likelihood (A1a) as:

(A4)

From equation (A4) we can determine the log-likelihood of the data under using the Expectation-Maximization (EM) algorithm estimates of and (see [51]). Similarly, we can determine the log-likelihood of the data under using the EM algorithm estimates of and . We comment that the estimates of may differ under the null and alternative hypotheses.

It follows from the equation (A4) that the log-likelihoods and (equation (A1a)) are completely determined by misclassification parameters , the true parameters , and sample counts (). In the previous sentence, (respectively, ) refers to the situation under the null (respectively alternative) hypothesis, where the terms in equation (A4) are replaced (respectively, not replaced) by . Our test of versusis a likelihood ratio test [72], which we call the *l*ikelihood *r*atio *t*est *a*llowing for *e*rror, or LRT_ae_. It is given by

LRT_ae_	(A5a)

where and are determined using equation (A4) with the EM-algorithm estimates of the various parameters. Asymptotically, LRT_ae_ is distributed as , where the degrees of freedom (df) are *k* – 1 for a set of *k* observed haplotype pairs (see [51]). For small samples or in situations where the asymptotic distribution may not hold, we can compute p-values via permutation (see [51,73]).

The standard likelihood ratio test, denoted LRT_std_, that does not make any correction, has its log-likelihoods computed solely from the observed data. That is,

LRT_std_	(A5b)

where the log-likelihoods under the null and alternative hypotheses are computed using the estimates, () that are then substituted into equation (A1b) [74]. When there is no correction for misclassification, there is no need to compute under both the null and alternative hypothesis, as the terms with will cancel from the difference of the log-likelihoods (equation (A5b)).

**Additional References**

72. Kendall M, Stuart A, Ord JK (1994) The Advanced Theory of Statistics. New York: Oxford University Press.

73. Barral S, Haynes C, Stone M, Gordon D (2006) LRTae: improving statistical power for genetic association with case/control data when phenotype and/or genotype misclassification errors are present. BMC Genet 7: 24.

74. Rice KM, Holmans P (2003) Allowing for genotyping error in analysis of unmatched cases and controls. Ann Hum Genet 67: 165-174.
